# Pyosalpinx-Induced Pelvic Sepsis Prompting Bilateral Salpingo-Oophorectomy and Laparoscopic Hysterectomy in a Premenopausal Woman

**DOI:** 10.7759/cureus.100158

**Published:** 2025-12-26

**Authors:** Rohan M Patel, Emily Forster, Tiffany S Sculthorpe, Kashif Abad

**Affiliations:** 1 Dr. Kiran C. Patel College of Osteopathic Medicine, Nova Southeastern University, Fort Lauderdale, USA; 2 Internal Medicine, Broward Health, Fort Lauderdale, USA

**Keywords:** antibiotic administration in management of severe sepsis, bacterial vaginosis (bv), bilateral salpingo-oophorectomy, non-sexually transmitted, pelvic inflammatory disease (pid), pelvic sepsis, pyosalpinx, source control, total laparoscopic hysterectomy, tubo-ovarian abscesses

## Abstract

Pyosalpinx is a rare infection of the fallopian tube characterized by pus accumulation and inflammation, which can progress to severe sepsis if not promptly treated. We describe a 54-year-old premenopausal woman who presented with fever, localized abdominal pain, and laboratory findings consistent with sepsis. Diagnostic imaging revealed a complex adnexal mass concerning for a tubo-ovarian abscess. Despite empiric broad-spectrum antibiotic therapy, the infection persisted, prompting a robotic-assisted total laparoscopic hysterectomy with bilateral salpingo-oophorectomy for definitive source control. Surgical pathology confirmed pyosalpinx with abscess formation. The presence of clue cells and the absence of sexually transmitted pathogens suggested bacterial vaginosis as the likely infectious source. This case emphasizes that upper genital tract infections can occur in women without traditional risk factors and that altered vaginal flora may play a significant role in their development. Prompt diagnosis, empiric antibiotic coverage, and timely surgical intervention remain crucial to prevent complications and ensure favorable outcomes.

## Introduction

Pyosalpinx is characterized by the purulent obstruction and distention of the fallopian tube, most commonly in the setting of pelvic inflammatory disease (PID). The infection ascends from the lower genital tract to the fallopian tubes, resulting in inflammation, damage to the endothelium, and edema. This ultimately contributes to tubal occlusion and pus accumulation [1,2]. Untreated infections can develop into tubo-ovarian abscesses and eventually progress to sepsis. Clinically, patients can present with lower abdominal pain, fever, tachycardia, and leukocytosis, findings consistent with a systemic inflammatory response [3]. According to the Sepsis-3 consensus definition, sepsis is identified as life-threatening organ dysfunction caused by a dysregulated host response to infection, operationalized as an acute increase of ≥ 2 points in the Sequential Organ Failure Assessment (SOFA) score [4]. 

Recent epidemiological data suggest that PID remains a significant global health concern despite improved sexually transmitted infection (STI) screening and widespread antibiotic use [5]. The prevalence of PID was estimated to be 53 per 100,000 in women of reproductive age, with declining trends observed in high-income countries [5]. A small subset of PID cases progress into pyosalpinx; one study reported that approximately 7.4% of hospitalized PID patients were found to have pyosalpinx on imaging or upon intra-operative evaluation [6]. Pyosalpinx typically presents in sexually active women of reproductive age with a history of *Chlamydia trachomatis* or *Neisseria gonorrhoeae*. However, pyosalpinx has been reported in the absence of STIs, often seen in polymicrobial infections including *Escherichia coli*, *Gardnerella vaginalis*, *Bacteroides fragilis*, and *Peptostreptococcus *[3,7].

Progression of pyosalpinx to sepsis is rare. Diagnosis can be challenging because the clinical presentation and imaging findings of pyosalpinx often resemble those of other adnexal or gastrointestinal conditions. This often leads to delayed management and a higher risk of complications, such as rupture, abscess formation, or peritonitis [8]. Surgical intervention is required in severe infections for definitive source control [7,8]. 

We present the case of a premenopausal woman with no known history of sexually transmitted infection who developed pyosalpinx complicated by sepsis. Despite broad-spectrum antibiotic therapy, her condition required surgical intervention with total hysterectomy and bilateral salpingo-oophorectomy. This case highlights the importance of early recognition, appropriate empiric coverage, and timely surgical management in preventing severe infectious complications. This case is particularly unusual given the patient’s premenopausal status, absence of predisposing sexually transmitted infection, and rapid progression to sepsis. 

## Case presentation

A 54-year-old female presented to the emergency department complaining of sudden-onset, sharp, intermittent lower abdominal pain for seven days before arrival. The patient also endorsed intermittent vaginal bleeding, nausea throughout the week, and one episode of vomiting. Her normal menstrual cycles usually occurred once monthly and lasted between four and five days. Her last menstrual period, however, lasted roughly 19 days. Upon emergency department arrival, the patient had a temperature of 39.4℃ (102.9℉) and her heart rate was 113 beats per minute. Abdominal exam revealed no diffuse peritoneal signs, but the left lower quadrant (LLQ) was tender to palpation with mild guarding. Pelvic exam revealed yellow discharge and left adnexal tenderness. The patient stated she was in a monogamous relationship with her husband and denied any history of sexually transmitted infection or disease. She also had no history of abnormal Pap examinations, with her most recent one being two years prior. Her gynecological history was notable for secondary infertility and two elective pregnancy terminations, at age 13 and age 16. In the past three months, the patient reported having increased her alcohol consumption to roughly 10 drinks per day, which she attributed to witnessing familial trauma. She had not had any alcoholic beverages for three weeks preceding admission, however. 

Urinalysis was performed (Table 1), which revealed an acute urinary tract infection, while a drawn complete blood count (Table 2) showed a leukocytosis. Based on the Sepsis-3 consensus definition, the patient met criteria for sepsis due to her heart rate, temperature, and leukocytosis. Blood cultures were negative. 

**Table 1:** Urinalysis revealing an acute urinary tract infection WBC: White blood cells, RBC: Red blood cells.

	Value	Reference Range
Color, Urine	Light-Orange	Yellow
Appearance, Urine	Extra Turbid	Clear
Glucose, Urine	Normal	Normal/Trace
Bilirubin, Urine (mg/dL)	Negative	Negative
Ketones, Urine (mg/dL)	Negative	Negative
Specific Gravity, Urine	1.023	1.015-1.025
Blood, Urine	Small	Negative
pH, Urine	5.5	5.0-7.5
Protein, Urine (mg/dL)	30	Negative
Leukocyte Esterase, Urine (leu/ul)	2	Negative
Nitrite, Urine	Negative	Negative
WBC, Urine	606	<= 5/HPF
RBC, Urine	19	<= 5/HPF
Mucus, Urine	Moderate	None seen
Squamous Epithelial, Urine	14	<= 10/HPF

**Table 2:** Complete blood count demonstrating a leukocytosis

	Value	Reference Range
WBC (10³/µL)	17.08	4.00-11.00
Neutrophils Absolute (10³/µL)	13.29	1.80-7.70
Red Blood Cells (10⁶/µL)	4.09	3.80-5.30
Hemoglobin (g/dL)	9.8	11.5-15.3
Hematocrit (%)	32.2	34.0-45.0
Platelets (10³/µL)	782	140-400

**Figure 1:**
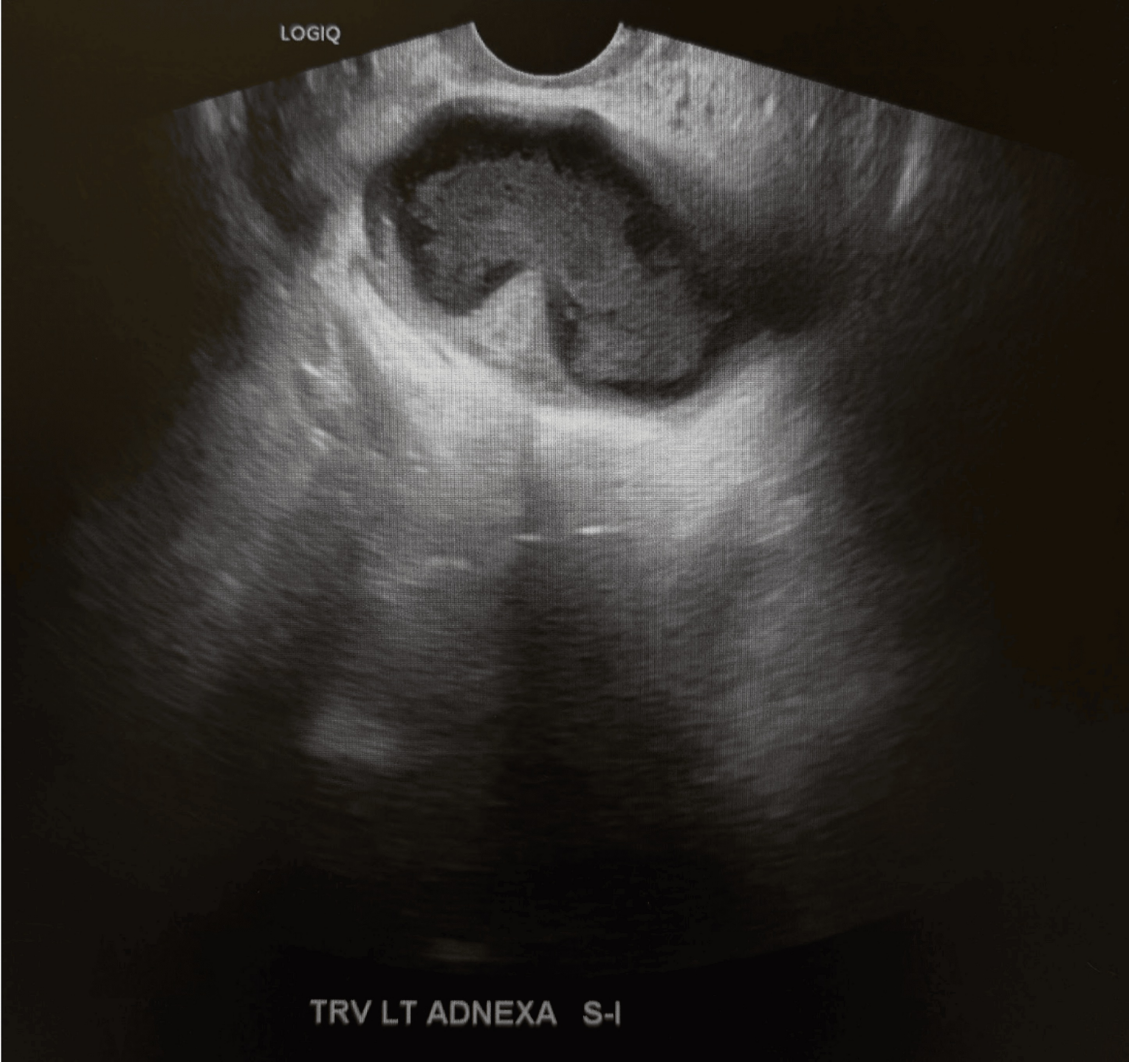
Transvaginal pelvic ultrasound with Doppler image Transvaginal pelvic ultrasound with Doppler showing a complex cystic structure with a tubular configuration containing material measuring 12 x 7.3 x 10 cm, suggestive of a potential hydrosalpinx or pyosalpinx.

A few clue cells were seen on the ordered trichomonas screen; however, no motile trichomonads were identified. Nucleic acid amplification tests (NAATs) for *Chlamydia trachomatis* and *Neisseria gonorrhoeae* were negative. At this time, the patient was placed on cefoxitin, doxycycline, and metronidazole, enabling broad-spectrum coverage for pelvic inflammatory disease. A chest radiograph was ordered secondary to her fever and depicted no acute cardiopulmonary process. A transvaginal pelvic ultrasound with Doppler was performed secondary to her pelvic pain, which revealed a complex cystic structure with a tubular configuration containing internal echogenic material measuring 12 x 7.3 x 10 cm, suggestive of a hydrosalpinx or pyosalpinx (Figure 1). 

**Figure 2:**
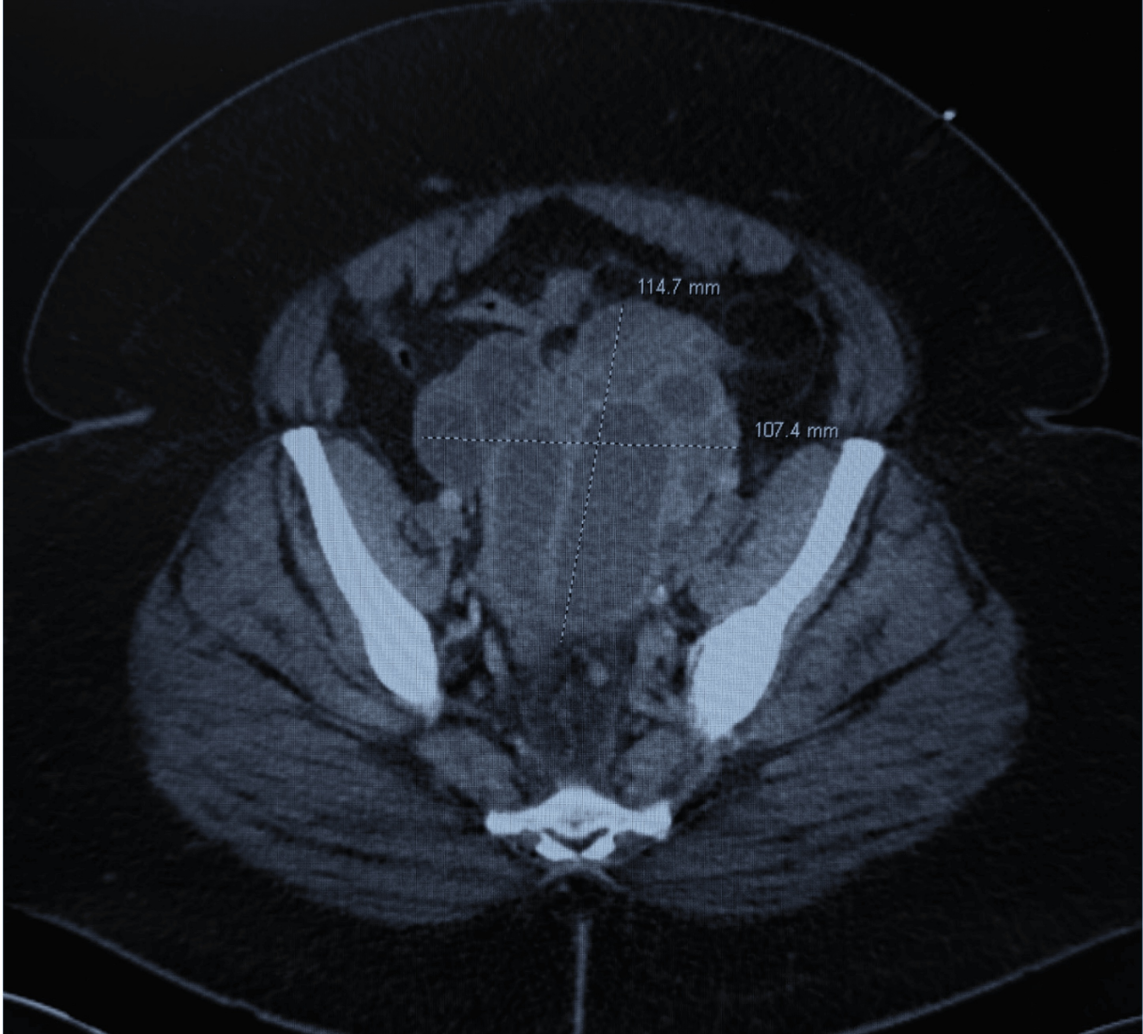
CT image of abdomen/pelvis CT abdomen/pelvis demonstrating a large complex septated mass in the pelvis containing multiple tubular structures consistent with adnexal origin.

A CT scan of the abdomen and pelvis subsequently revealed a large, complex, septated pelvic mass containing multiple tubular structures consistent with adnexal origin (Figure 2). Additionally, abnormally enlarged retroperitoneal and left common iliac chain lymph nodes were noted. At that time, malignancy could not have been excluded; however, her clinical presentation was more suggestive of an acute infectious process. A repeat CBC obtained the following day demonstrated a persistent leukocytosis, with a white blood cell (WBC) count of 14.41 × 103/µL, indicative of an initial response to antibiotic therapy. 

The patient was diagnosed with a tubo-ovarian abscess. After consultation with interventional radiology, a plan was made to proceed with a percutaneous drainage due to the presence of purulent vaginal discharge. However, the mass was ultimately determined to be inaccessible secondary to the patient’s body habitus. Gynecologic oncology was therefore consulted for surgical evaluation. Following discussion of the available management options, the patient expressed a desire for definitive treatment and elected to proceed with a total hysterectomy and bilateral salpingo-oophorectomy. 

The patient underwent a robotic-assisted total laparoscopic hysterectomy with bilateral salpingo-oophorectomy that afternoon. Extensive adhesiolysis was required between the anterior abdominal wall and small bowel to adequately visualize the uterus. Large abscess cavities, densely adherent to the posterior cul-de-sac structures, were identified bilaterally. The excised uterus weighed 300 grams, consistent with a fibroid uterus. On postoperative day one, the patient had a WBC count of 12.33 × 103/µL. The surgical pathology report demonstrated ectocervical and endocervical mucosa with squamous metaplasia, without evidence of dysplasia or malignancy. Myometrial tissue revealed an intramural leiomyoma without cellular atypia. The ultimate pathologic diagnosis was a pyosalpinx, characterized by marked suppurative acute inflammation and abscess formation. The patient’s final complete blood count (CBC) showed a WBC count within normal limits. She was discharged home in stable condition with instructions to continue antibiotics for an additional 14 days. 

## Discussion

This case highlights a rare presentation of pyosalpinx rapidly progressing to sepsis and ultimately necessitating surgical management. In contrast to the traditional presentation of pyosalpinx in the context of PID, this patient exhibited a polymicrobial infection, suggesting a distinct pathophysiologic mechanism of inoculation. Notably, severe upper gynecological infections can occur in women without traditional risk factors, particularly in the setting of temporary immunosuppression or altered vaginal flora. 

Several contributing factors may have led to the progressive sepsis observed in this patient. The presence of clue cells in the absence of trichomonads or other sexually transmitted pathogens suggests inoculation of *Gardnerella vaginalis*, a recognized cause of tuboovarian abscesses [9]. Although bacterial vaginosis is not classified as an STI, it alters the chemical environment of vaginal flora, allowing for the overgrowth of anaerobic bacteria [10]. This manipulated microenvironment enables pathogenic ascension from the lower to upper genital tract, predisposing to PID and, as seen here, likely contributing to the development of a tubo-ovarian abscess. Furthermore, recent psychosocial stressors, coupled with alcohol-induced impairment of mucosal immunity, could have facilitated an ascending genitourinary infection through the inability of pro-tumor necrosis factor (TNF)α to convert into its active form, thereby weakening the innate immune response [11].

Diagnosing adnexal infections presents inherent challenges. Ambiguous imaging findings often prompt clinicians to consider a broad differential diagnosis requiring further evaluation [12]. In this patient, the transvaginal ultrasound initially raised concern for a pyosalpinx or hydrosalpinx; although CT imaging demonstrated a complex mass with lymphadenopathy, raising concern for malignancy, this was less likely given her clinical presentation. The diagnostic uncertainty associated with adnexal pathologies delineates the importance of taking a multifaceted approach to gynecologic evaluation. Correlating laboratory data with clinical findings while integrating radiologic and gynecologic oncology expertise to obtain a comprehensive, evidence-based perspective is paramount. Given the patient’s initial presentation, management with cefoxitin, doxycycline, and metronidazole provided broad-spectrum empiric coverage consistent with the Centers for Disease Control and Prevention guidelines for inpatient PID [13]. However, the patient’s leukocytosis and fever persisted. Due to the limitations of percutaneous access, the clinical discussion shifted toward definitive surgical source control. Considering the patient’s septic status, prompt intervention was warranted. According to the Journal of Intensive Medicine, the timing of source control significantly impacts prognosis, particularly in cases involving intra-abdominal infections; in fact, adequate source control is a stronger prognostic indicator than the specific mode of intervention utilized [14]. Surgical intervention in this case resulted in complete resolution of infection and an expeditious recovery, supporting the assertion that early surgical intervention in refractory pyosalpinx cases improves clinical outcomes [15].

## Conclusions

This case broadens the recognized clinical spectrum of pyosalpinx by demonstrating its occurrence and rapid progression to sepsis in a premenopausal woman with no history of STI. Clinicians should maintain a high index of suspicion for pyosalpinx in patients presenting with adnexal pain and imaging findings consistent with complex pelvic masses, even in the absence of classic risk factors. A multidisciplinary approach, prompt empiric antibiotic coverage, and timely surgical source control remain critical in achieving favorable patient outcomes. 
